# Genome Sequence of Luminous Piezophile *Photobacterium phosphoreum* ANT-2200

**DOI:** 10.1128/genomeA.00096-14

**Published:** 2014-04-17

**Authors:** Sheng-Da Zhang, Valérie Barbe, Marc Garel, Wei-Jia Zhang, Haitao Chen, Claire-Lise Santini, Dorothée Murat, Hongmei Jing, Yuan Zhao, Aurélie Lajus, Séverine Martini, Nathalie Pradel, Christian Tamburini, Long-Fei Wu

**Affiliations:** a Deep-Sea Research Department, Sanya Institute of Deep-Sea Science and Engineering, Chinese Academy of Sciences, Sanya, China; b Laboratoire de Chimie Bactérienne, Aix-Marseille Université, Centre National de la Recherche Scientifique, Unité Mixte de Recherche 7283, Marseille, France; c France-China Bio-Mineralization and Nano-Structure Laboratory (LIA-BioMNSL), Sanya Institute of Deep-Sea Science and Engineering of the Chinese Academy of Sciences, Sanya, China; d DSV/IG/Genoscope/LF, Évry, France; e Aix-Marseille Université, Université du Sud Toulon-Var, CNRS/INSU, IRD, Mediterranean Institute of Oceanography (MIO), UM110, Marseille, France; f Key Laboratory of Marine Ecology & Environmental Sciences, Institute of Oceanology, Chinese Academy of Sciences, Qingdao, China; g CEA/DSV/IG/Genoscope & CNRS-UMR 8030 & University of Évry Val d'Essone, Laboratoire d’Analyse Bioinformatique en Génomique et Métabolisme, Évry, France

## Abstract

Bacteria of the genus *Photobacterium* thrive worldwide in oceans and show substantially varied lifestyles, including free-living, commensal, pathogenic, symbiotic, and piezophilic. Here, we present the genome sequence of a luminous, piezophilic *Photobacterium phosphoreum* strain, ANT-2200, isolated from a water column at 2,200 m depth in the Mediterranean Sea. It is the first genomic sequence of the *P. phosphoreum* group. An analysis of the sequence provides insight into the adaptation of bacteria to the deep-sea habitat.

## GENOME ANNOUNCEMENT

*Photobacterium* is one of the major genera of the family *Vibrionaceae* and is widespread in marine environments. Previously, the name *Photobacterium phosphoreum* was used to refer to various species. Recent multilocus fine-scale phylogenetic analysis has resolved three distinct species in the *P. phosphoreum* group: *Photobacterium phosphoreum*, *Photobacterium iliopiscarium*, and *Photobacterium kishitanii* (1). Among them, *P. phosphoreum* and *P. kishitanii* are luminous, and *P. kishitanii* is the only species found in the light organs of deep-sea fishes. None of the *P. iliopiscarium* strains analyzed emit light. *P. phosphoreum* ANT-2200 is a piezophilic species isolated from the Mediterranean Sea at a depth of 2,200 m (2, 3). To gain insight into the genetic traits underlying the differences among the three closely related species of the *P. phosphoreum* group dwelling in different environments, we sequenced the genome of *P. phosphoreum* ANT-2200.

The ANT-2200 whole-genome shotgun sequence was obtained using the Illumina technology. Mate-paired (MP) and paired-end (PE) libraries were created with 8-kb and 350-bp insert sizes, respectively. The sequencing was performed on a MiSeq instrument with paired read lengths of 150, leading to 90- and 180-fold coverage for the MP and PE data, respectively. These data were assembled using Velvet 1.0 (http://www.ebi.ac.uk/~zerbino/velvet), and to reduce the number of undetermined bases, GapCloser (http://soap.genomics.org.cn/soapdenovo.html) was used on the scaffold sequences, with the PE reads. The annotation was performed using the MicroScope platform (https://www.genoscope.cns.fr/agc/microscope/home/index.php) (4).

The whole-genome draft of *P. phosphoreum* ANT-2200 consists of 25 contigs assembled into 19 scaffolds, for a total of 5,107,216 bp with 38.89% G+C content. A total of 4,667 coding DNA sequences were predicted, as well as 36 miscellaneous RNAs, 6 disrupted rRNA operons, and 168 tRNA genes.

Among the NCBI genomic reference sequences, the top 11 that were homologous to the *P. phosphoreum* ANT-2200 genome are from 6 *Photobacterium* genomes with decreasing *E* values, in the following order: *P. leiognathi* subsp. *mandapamensis* svers.1.1 (accession no. NZ_DF093598.1), *P. leiognathi* lrivu.4.1 (accession no. NZ_DF196819.1), *P. angustum* S14 (accession no. NZ_CH902602.1), *P. profundum* SS9 (accession no. NC_006370.1), *Photobacterium* sp. strain SKA34 (accession no. NZ_CH724145.1), *P. angustum* S14 (accession no. NZ_CH902604.1), *P. leiognathi* subsp. *mandapamensis* svers.1.1 (accession no. NZ_DF093594.1), *P. leiognathi* lrivu.4.1 (accession no. NZ_DF196811.1), *Photobacterium* sp. SKA34 (accession no. NZ_CH724147.1), *P. angustum* S14 (accession no. NZ_CH902603.1), and *P. profundum* 3TCK (accession no. NZ_CH724136.1). A synton of the *luxLCDABFEG-ribEBHA* luminous genes was found in the ANT-2200 genome and is perfectly conserved in the genome of *P. leiognathi* subsp. *mandapamensis* svers.1.1. As a trait of adaptation to the deep-sea habitat, the genome of the piezophilic *P. profundum* strain SS9 contains 2 sets of flagellar genes functioning under different pressures and viscosities (5). ANT-2200 bears two sets of flagellar genes that are highly conserved compared to those from SS9. Furthermore, SS9 has multiple *torA* genes encoding trimethylamine *N*-oxide reductases, one of them being upregulated by high hydrostatic pressure (6). Interestingly, the genome of *P. phosphoreum* ANT-2200 has four copies of *torA*, including the counterpart of the pressure-upregulated *torA*. The genome of *P. phosphoreum* ANT-2200 will be used as the first representative of the *P. phosphoreum* group in studies of the evolution of luminous bacteria and adaptation of bacteria to the deep-sea habitats.

### Nucleotide sequence accession numbers.

The genome sequence of *P. phosphoreum* ANT-2200 was deposited in EMBL under accession no. CCAR010000001 to CCAR010000025 .

## References

[1] Ast JC Dunlap PV 2005 Phylogenetic resolution and habitat specificity of members of the *Photobacterium phosphoreum* species group. Environ. Microbiol. 7:1641–1654. 10.1111/j.1462-2920.2005.00859.x 16156737

[2] Al Ali B Garel M Cuny P Miquel JC Toubal T Robert A Tamburini C 2010 Luminous bacteria in the deep-sea waters near the ANTARES underwater neutrino telescope (Mediterranean Sea). Chem. Ecol. 26:57–72. 10.1080/02757540903513766

[3] Martini S Al Ali B Garel M Nerini D Grossi V Pacton M Casalot L Cuny P Tamburini C 2013 Effects of hydrostatic pressure on growth and luminescence of a moderately-piezophilic luminous bacteria *Photobacterium phosphoreum* ANT-2200. PLoS One 8:e66580. 10.1371/journal.pone.0066580 23818946 PMC3688590

[4] Vallenet D Belda E Calteau A Cruveiller S Engelen S Lajus A Le Fèvre F Longin C Mornico D Roche D Rouy Z Salvignol G Scarpelli C Thil Smith AA Weiman M Médigue C 2013 Microscope—an integrated microbial resource for the curation and comparative analysis of genomic and metabolic data. Nucleic Acids Res. 41:D636–D647. 10.1093/nar/gks1194 23193269 PMC3531135

[5] Eloe EA Lauro FM Vogel RF Bartlett DH 2008 The deep-sea bacterium *Photobacterium profundum* SS9 utilizes separate flagellar systems for swimming and swarming under high-pressure conditions. Appl. Environ. Microbiol. 74:6298–6305. 10.1128/AEM.01316-08 18723648 PMC2570297

[6] Vezzi A Campanaro S D’Angelo M Simonato F Vitulo N Lauro FM Cestaro A Malacrida G Simionati B Cannata N Romualdi C Bartlett DH Valle G 2005 Life at depth: *Photobacterium profundum* genome sequence and expression analysis. Science 307:1459–1461. 10.1126/science.1103341 15746425

